# Clinical epigenetics and restoring of metabolic health in severely obese patients undergoing batriatric and metabolic surgery

**DOI:** 10.1007/s13304-021-01162-9

**Published:** 2021-10-02

**Authors:** Mario Faenza, Giuditta Benincasa, Ludovico Docimo, Giovanni Francesco Nicoletti, Claudio Napoli

**Affiliations:** 1 Multidisciplinary Department of Medical, Surgical and Dental Sciences, Plastic Surgery Unit, University of Campania “Luigi Vanvitelli”, Naples, Italy; 2 Department of Advanced Medical and Surgical Sciences (DAMSS), University of Campania “Luigi Vanvitelli”, Naples, Italy; 3 Division of General, Mininvasive and Bariatric Surgery, University of Campania “Luigi Vanvitelli”, Via Pansini 5, 80100 Naples, Italy; 4 Clinical Department of Internal Medicine and Specialistics, Division of Clinical Immunology, Transfusion Medicine and Transplant Immunology, AOU University of Campania “Luigi Vanvitelli”, Naples, Italy

**Keywords:** Obesity, Bariatric and metabolic surgery, Epigenetics, Precision medicine

## Abstract

Epigenetic-sensitive mechanisms, mainly DNA methylation, mirror the relationship between environmental and genetic risk factors able to affect the sensitiveness to development of obesity and its comorbidities. Bariatric and metabolic surgery may reduce obesity-related cardiovascular risk through tissue-specific DNA methylation changes. Among the most robust results, differential promoter methylation of *ACACA*, *CETP*, *CTGF*, *S100A8*, and *S100A9* genes correlated significantly with the levels of mRNA before and after gastric bypass surgery (RYGB) in obese women. Additionally, promoter hypermethylation of *NFKB1* gene was significantly associated with reduced blood pressure in obese patients after RYGB suggesting useful non-invasive biomarkers. Of note, sperm-related DNA methylation signatures of genes regulating the central control of appetite, such as *MC4R*, *BDNF*, *NPY*, and *CR1*, and other genes including *FTO*, *CHST8*, and *SH2B1* were different in obese patients as compared to non-obese subjects and patients who lost weight after RYGB surgery. Importantly, transgenerational studies provided relevant evidence of the potential effect of bariatric and metabolic surgery on DNA methylation. For example, peripheral blood biospecimens isolated from siblings born from obese mothers before bariatric surgery showed different methylation signatures in the insulin receptor and leptin signaling axis as compared to siblings born from post-obese mothers who underwent surgery. This evidence suggests that bariatric and metabolic surgery of mothers may affect the epigenetic profiles of the offspring with potential implication for primary prevention of severe obesity. We update on tissue-specific epigenetic signatures as potential mechanisms underlying the restoration of metabolic health after surgery suggesting useful predictive biomarkers.

## Introduction

Despite the efforts in discovering novel potential non-invasive biomarkers, we are unable to stratify the risk of recovering the weight lost and developing cardiovascular diseases (CVDs) in severely obese patients undergoing weight change [1, 2]. Obesity has globally reached epidemic proportions, increasing the risk for type 2 diabetes (T2D) and CVDs as major causes of morbidity and mortality worldwide [3]. Advanced epigenomic-based technologies are providing novel insight into the pathogenesis of obesity and CVDs [3–7]. Direct epigenetic marks consist in DNA methylation and histone modification changes occurring as a response to specific environmental exposures, which can affect gene expression programs without modifications in genetic background [8]. Epigenetic-sensitive mechanisms can be acquired over the lifetime leading to chromatin remodeling and, therefore, alterations of transcriptional programs underlying oxidative stress, inflammation, and metabolic unbalance which may predispose to obesity [4–8].

Bariatric and metabolic surgery is an effective therapy in the management of severe obesity and related cardiometabolic risk [9–12], and, generally, is suggested to patients with a body mass index (BMI) ≥ 40 kg/m [2] who have failed traditional nonsurgical approaches [13–15]. Chronologically, we had Roux-en-Y gastric bypass (RYGB), biliopancreatic diversion (BPD), duodenal switch (DS), adjustable gastric banding (AGB), and sleeve gastrectomy (SG). RYGB, BPD, and DS dominated the scene as gold standards in the surgical treatment of patients with severe obesity from the 1960s to the 2000s and have been replaced by SG. The reason for this change resides in the lower rate of complications both in the short (anastomotic leaks, small bowel obstruction, staple-line leaks, hemorrhage) and long term (anastomotic strictures, marginal ulceration, gastric fistula, nutritional deficiencies, liver failure, and recurrent weight gain) and in the less complexity of the whole surgical procedure [13–15]. In the early 2000s, laparoscopic AGB started to have considerable appeal both for surgeons and patients, given the simple technique and low short-term complication rate; however, the cluster of patients in which this type of procedure was indicated rapidly diminished owing to weight regain and long-term risk of band perforation/slippage [13–15]. SG has the advantage of not requiring any bowel manipulation or anastomosis, but there are no data in the long term and the rate of staple-line leaks in the fundus of the sleeve and gastroesophageal reflux are still high.

The abnormalities of epigenetic-sensitive pathways, mainly guided from DNA methylation, in obese patients undergoing bariatric and metabolic surgery make the epigenetic information potentially useful for diagnostic and therapeutic strategies. Several longitudinal studies demonstrated that bariatric and metabolic surgery may reverse the obesity-related epigenome, mainly through changes in DNA methylome, suggesting that the epigenetic regulation may mediate the surgery-induced beneficial effects [16–21]. Thus, bariatric and metabolic surgery may serve as “epigenetic shaping” able to restore the metabolic balance in patients affected by severe obesity. The goal of this scoping review is to highlight the relevance of clinical epigenetics as new tool to be employed for clarifying molecular basis of obesity as well as to provide novel biomarkers useful for personalized management of patients after surgical intervention.

## Methods

We performed electronic searches in three different databases, including PubMed, Google Scholar, and Web of Science, to select only studies in patients evaluating the potential clinical role of DNA methylation changes in restoring metabolic health after surgical procedures. We searched for studies written in English with a priority for those published in the last 10 years. The search syntax consisted of terms related to “obese patients” and “metabolic and bariatric surgery” combined with terms “DNA methylation”, “peripheral blood”, “tissue biopsy”, and “biomarkers”. Two expert researchers in the field screened independently all studies and categorized them according to the size of the study population and type of human biospecimen. The search yielded 12 original articles evaluating the modifications of DNA methylome in obese patients before and after metabolic and bariatric surgery. Concerning the type of human biospecimen for DNA methylome mapping, five studies used whole blood, two studies used adipose tissue biopsy, three studies used skeletal muscle biopsy, one study used liver biopsy, and one study used spermatozoa.

## DNA methylation basic mechanisms

**Fig. 1:**
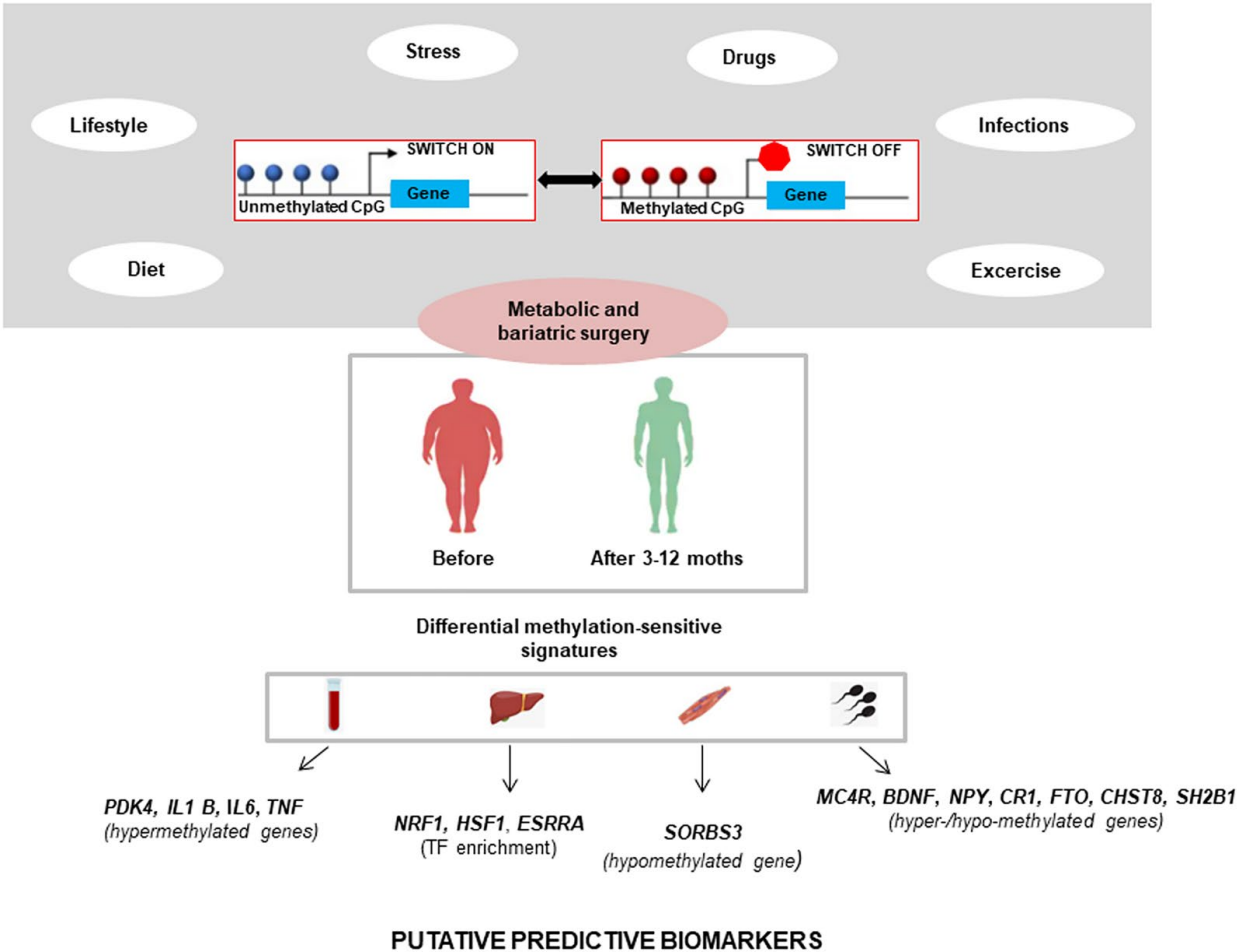
DNA methylation as potential mediator of bariatric and metabolic surgery effects in patients with severe obesity. DNA methylation changes in targeted genes may mirror the beneficial effects of surgery procedures on the weight lost. If mechanistically validated, these molecular signatures may represent useful non-invasive biomarkers to stratify the risk of weight recovery and cardiovascular complications

DNA methylation levels are modulated by environmental and lifestyle risk factors representing potential pathogenic mechanisms underlying metabolic alterations in patients predisposed to or affected by cardiometabolic diseases [4–6, 22]. DNA methylation is the biochemical addition of a methyl group which mainly occurs in the CpG dinucleotides through the action of DNA methyltransferase enzymes (DNMTs) [8]. The genomic loci with a high density of CpG dinucleotides [known as “CpG islands”, (GCIs)] are mainly located in the gene promoter regions. Generally, CGI methylation status inversely correlates with the rates of transcription regulation; in fact, promoter-related methylated CGIs are located upstream of silenced genes, whereas unmethylated CGIs are located upstream of transcriptionally active genes (Fig. 1) [8]. In contrast, methylation of intragenic regions including gene body untranslated regions (5’ and 3’ UTRs) seems to be related to increased gene expression [23]. Interestingly, baseline DNA methylation patterns were indicated as possible non-invasive biomarkers which could help to predict weight loss, but advance in this field is still limited to the bench of research laboratory [24].

## Obesity-related DNA methylation changes can be erased after metabolic and bariatric surgery

Beyond the weight loss, bariatric and metabolic surgery can induce several beneficial effects on metabolism including improvement of insulin sensitivity and cardiovascular function as well as resolving T2D [9, 25]. Specific molecular pathways, including chronic low-grade inflammation, release of incretin hormones, and lipid oxidation and mitochondrial function, seem to be improved after bariatric surgery. [26–29] Even if the molecular routes are still unclear, bariatric and metabolic surgery could restore the metabolic health by means of epigenetic regulation (Fig. 1). In particular, some studies have detected differences in DNA methylation levels upon specific types of bariatric surgery (Table 1). We summarize the most recent clinical evidence for which DNA methylation could mediate the beneficial effects of bariatric and metabolic surgery in specific patient-derived specimens.

**Table 1:** Examples of DNA methylation changes associated with restoring metabolic health after bariatric and metabolic surgery

Sample size	Platform	Genomic localization	Validation and RNA expression	Results	References
Adipose tissue					
Subcutaneous adipose and omentum tissues from 15 obese women before and after RYGB	HumanMethylation 450k BeadChip	Mainly untranslated regions and gene body	Pyrosequencing, qRT-PCR	Differential promoter methylation of *ACACA*, *CETP*, *CTGF*, *S100A8*, and *S100A9* genes correlated significantly with different levels of mRNA before and after RYGB	[18]
Abdominal subcutaneous fat from 16 post-obese women, 2 years after RYGB vs 14 never-obese women	Infinium HumanMethylation 450 BeadChip assay	Mainly at 5′ untranslated regions and gene bodies	Gene 1.1 ST Arrays	Global CpG hypomethylation and enrichment of adipogenesis genes in fat cells may contribute to adipose hyperplasia in post-obese women	[31]
Skeletal muscle					
Obese women before and 6 months after RYGB surgery vs 16 age-matched non-obese controls	Methyl-CpG binding protein-based system	Promoter non-CpG sites	Bisulfite sequencing analysis, qRT-PCR	Promoter methylation of *PGC-1α* and *PDK4* genes is altered with obesity and restored to non-obese levels after RYGB-induced weight loss	[20]
7 morbidly obese female subjects pre- and 3 months post-RYGB surgery	RRBS	Promoter and untranslated regions	Pyrosequencing, qRT-PCR	Hypomethylation of *SORBS3* gene was associated with an increase in gene expression and strongly correlated with fasting plasma glucose levels	[17]
49 obese Caucasian patients (OB) before and 2, 12, 24, and 52 weeks after SG or RYGB	Infinium^®^ MethylationEPIC BeadChip	Intergenic regions and gene body	ArrayXS Human	Surgically induced weight loss may modify DNA methylation of genes involved in muscle energy metabolism and associate with changes in gene expression along with restoration of muscle metabolism within 1 year	[16]
Peripheral blood					
11 obese patients both before and 6 months after RYGB surgery vs 16 normal-weight men	Illumina 450 K methylation chip	Promoter	–	RYGB decreased the genome-wide pre-surgery distance between promoter-specific DNA methylation in whole blood of obese patients vs controls	[37]
18 obese non-diabetic patients undergoing a 14 day VLCD, followed by RYGB vs 6 non-obese patients	Illumina 450 K methylation chip	Promoter	–	Methylation levels increased in *PDK4*, *IL1 B, IL6*, and *TNF* gene promoters 12 months after RYGB	[38]
60 severely obese patients examined before and at 6 months after bariatric surgery	Pyrosequencing	Promoter	–	*NFKB1* gene promoter hypermethylation was significantly associated with reduced blood pressure after surgery	[39]
A sibling cohort of 31 BMS vs 31 AMS offspring	Infinitum Human Methylation 450 BeadChip	Promoter and gene body	–	Bariatric surgery was associated with alterations in the methylome of genes involved in insulin receptor signaling, T2D, and leptin signaling in BMS vs AMS	[40]
A sibling cohort of 25 BMS vs 25 AMS offspring	Infinium HumanMethylation450 BeadChip	Gene body, untranslated regions, regulatory regions	HumanHT-12 v4 Expression BeadChip	IL8 pathway-related gene methylation correlated with both gene expression and PCR levels	[41]
Liver					
45 obese patients with all stages of NAFLD undergoing RYGB vs 18 controls	HumanMethylation450k Bead Chip	Regulatory regions	Pyrosequencing, HuGene 1.1 ST gene	Post-bariatric and NAFLD-specific methylation signatures was observed in *NRF1, HSF1*, and *ESRRA* genes	[35]
Spermatozoa					
13 obese men undergoing RYGB vs 10 controls	RRBS	Promoter, body and intergenic regions	Pyrosequencing	Sperm methylome was altered after RYGB in morbidly obese men at level of genes regulating the control of appetite (*MC4R*, *BDNF*, *NPY*, *CR1*) and metabolism (*FTO, CHST8, SH2B1*)	[36]

*ACACA* acetyl-coA carboxylase alpha, *AMS* siblings born after, *BDNF* brain-derived neurotrophic factor, *BMS* siblings born before, *CETP* cholesteryl ester transfer protein, *CHST8* carbohydrate sulfotransferase 8, *CR1* cannabinoid receptor type 1, *CTGF* connective tissue growth factor, *ESRRA* estrogen-related receptor alpha; *FTO* fat mass and obesity associated; *HSF1* heat shock TF 1, *IL1 B* interleukin 1B, *IL6* interleukin-6, *MC4R* melanocortin-4 receptor, *NAFLD* nonalcoholic fatty liver disease, *NFKB1* nuclear factor kappa b subunit 1, *NPY* neuropeptide Y, *NRF1* nuclear respiratory factor 1, *PDK4* pyruvate dehydrogenase kinase 4, *PDK4* pyruvate dehydrogenase lipoamide kinase isozyme 4, *PGC1A* proliferator-activated receptor gamma coactivator 1-alpha, *RRBS* reduced representation bisulfite sequencing, *RYGB* gastric bypass surgery, *S100A8* S100 calcium-binding protein A8, *S100A9* S100 calcium-binding protein A9, *SG* sleeve gastrectomy, *SH2B1* SH2 binding domain containing protein 1, *TNF* tumor necrosis factor, *VLCD* very low calorie diet, *PCR* plasma C-reactive protein, *IL8* interleukin 8

### Adipose tissue

The acceleration of visceral adipose tissue (VAT)-related epigenetic age was measured by hypermethylation of specific CpGs and seemed to have potential effects on the process of body weight loss after bariatric and metabolic surgery [30]. Adipose tissue and its DNA methylation profiles have a key role in obese patients [18]. Benton et al. [18] found a cluster of differentially methylated genes in the subcutaneous and omental adipose tissues isolated from obese women before and after an RYGB. These genes were functionally associated with obesity, epigenetic regulation, and development process and showed overlapping modifications in their transcriptional profiles, thus remarking the impact of DNA methylation across surgery procedures [18]. Besides, Dahlman et al. [31] demonstrated that a specific panel of genes involved in adipogenesis was differentially methylated in their regulatory regions in abdominal subcutaneous fat cells isolated from post-obese women with respect to healthy controls. However, modifications in the related transcriptional profiles were not found [31].

### Skeletal muscle

To date, there is no evidence for the potential effects of RYGB surgery on the global DNA methylation in human tissues, such as muscle biopsy specimens [20, 32]. The methylation levels of the long interspersed nuclear element 1 (LINE-1), considered as a marker of global DNA methylation, did not show changes after RYGB-induced weight loss [33, 34]. Otherwise, RYGB surgery was significantly associated with targeted modifications in DNA methylation levels of some genes, such as the peroxisome proliferator-activated receptor gamma coactivator 1-alpha (*PGC1A*) and pyruvate dehydrogenase lipoamide kinase isozyme 4 (*PDK4*), which are involved in the regulation of lipid metabolism in skeletal muscle [20]. After a period of 6 months, modifications in DNA methylation levels were also associated with variations in mRNA levels of these genes, suggesting a potential role in restoring metabolic health after surgery [20]. The RYGB-induced weight loss restored to normal levels both the sorbin and SH3 domain containing 3 (*SORBS3*) promoter methylation levels and gene expression in the muscle specimens of obese female [17]. In addition, post-surgery (at 3 months) changes in the *SORBS3* mRNA levels correlated with obesity-related parameters and fasting insulin levels, suggesting molecular routes which were potentially involved in the restoration of metabolic health after surgery procedures [17]. Recently, an integrated omic-based approach has demonstrated that significant changes in DNA methylation mostly occurred both in inter- and intragenic regions at 52 weeks (not early) from the RYGB procedure, affecting the expression profiles of genes involved in mitochondrial, lipidic and calcium-related signaling axes [16].

### Liver and spermatozoa

A case–control study showed an alteration in DNA methylation/transcriptional profiles in liver biopsies isolated from patients with obesity and complicated by different grades of non-alcoholic fatty liver disease (NAFLD) as compared to patients undergoing bariatric surgery [35]. In particular, nine genes encoding for the main enzymes regulating the intermediate metabolism seem to mediate dynamic remodeling, which is induced after the massive weight loss after bariatric surgery [35]. For the first time, sperm biospecimens were collected at three time points from men grouped in three classes: (1) healthy (BMI 20–25), (2) obesity (BMI > 29), and severe obesity (BMI > 40) to study whether and how dynamic changes of DNA methylation may have a mechanistic role in obesity [36]. Sperm-related DNA methylation changes mirrored a potential direct involvement of genes regulating the central control of appetite and metabolic processes in obese men as compared to men who lost weight after RYGB suggesting potential biomarkers to longitudinally trace the effect of surgery procedures. [36].

Targeted DNA methylation signatures were detected in blood leukocytes and showed no substantial changes in patients with obesity as compared to healthy controls [37]. Interestingly, Kirchner et al. [38] found that 12 months after RYGB surgery, specific promoter regions annotated to the interleukin (IL)-6,IL-1B, tumor necrosis factor α (TNF-α), and pyruvate dehydrogenase kinase 4 (PKD4) genes were hypermethylated suggesting a putative mechanistic link between methylation profiles and beneficial effect of bariatric surgery. Interestingly, promoter hypermethylation of the nuclear factor kappa b subunit 1 (*NFKB1*) gene was significantly associated with reduced blood pressure after surgery suggesting useful non-invasive biomarkers [39].

The potential effects of bariatric and metabolic surgery on DNA methylation are supported by multigenerational studies conducted on obese women before and after bariatric surgery and their offspring. Siblings born from obese mothers before BPD surgery showed different methylation signatures as compared to siblings born from post-obese mothers who underwent surgery. This supports the idea for which the surgical procedure of mothers may modify the epigenetic profiles in the offspring, and, in consequence, the risk of developing cardiovascular complications in adulthood. At molecular level, differentially methylated regions were annotated to insulin receptor and leptin signaling axis in obesity [40]. Besides, the maternal surgical treatment induced differences in the DNA methylation/transcriptional profiles of pro-inflammatory genes in children as compared to their siblings who were conceived pre-surgery [41]. In particular, DNA methylation and transcriptional levels of IL-8-related genes correlated with plasma C-reactive protein levels suggesting useful cardiometabolic risk biomarkers.

## Concluding remarks

Despite that clinical epigenetics is a promising tool for management of cardiometabolic diseases [42–46], the ability to predict outcomes of patients after metabolic and bariatric surgery though DNA methylation changes is still in its infancy. Clinical studies conducted so far are few and small in sample size and performed on different sequencing techniques to map DNA methylome. These non-standardized research protocols did not allow to obtain robust results which would direct the attention toward a biomarker rather than another. Another challenge related to the study of DNA methylation and restoring of metabolic health is the demonstration of a causal–effect relationship between these two factors. One of the most advanced tools to evaluate the presumed causal role of DNA methylation changes in obesity and CVDs is network medicine, which combine advanced omics platforms, potent bioinformatic algorithms, and clinical information [47–49]. Thus, more large longitudinal studies should be conducted to define whether the epigenome profiled before and after metabolic and bariatric surgery may help clinicians in predicting outcomes of severe obese patients.
